# Supplementary material for: Complex‐centric proteome profiling by SEC‐SWATH‐MS
Source: Mol Syst Biol. 2019 Jan 14;15(1):e8438. doi: 10.15252/msb.20188438 (PMC6346213; doi:10.15252/msb.20188438)
Supplement: Supplementary file 8 — Dataset EV7 [file MSB-15-e8438-s008.zip › feature_plots_string/P27708.pdf]

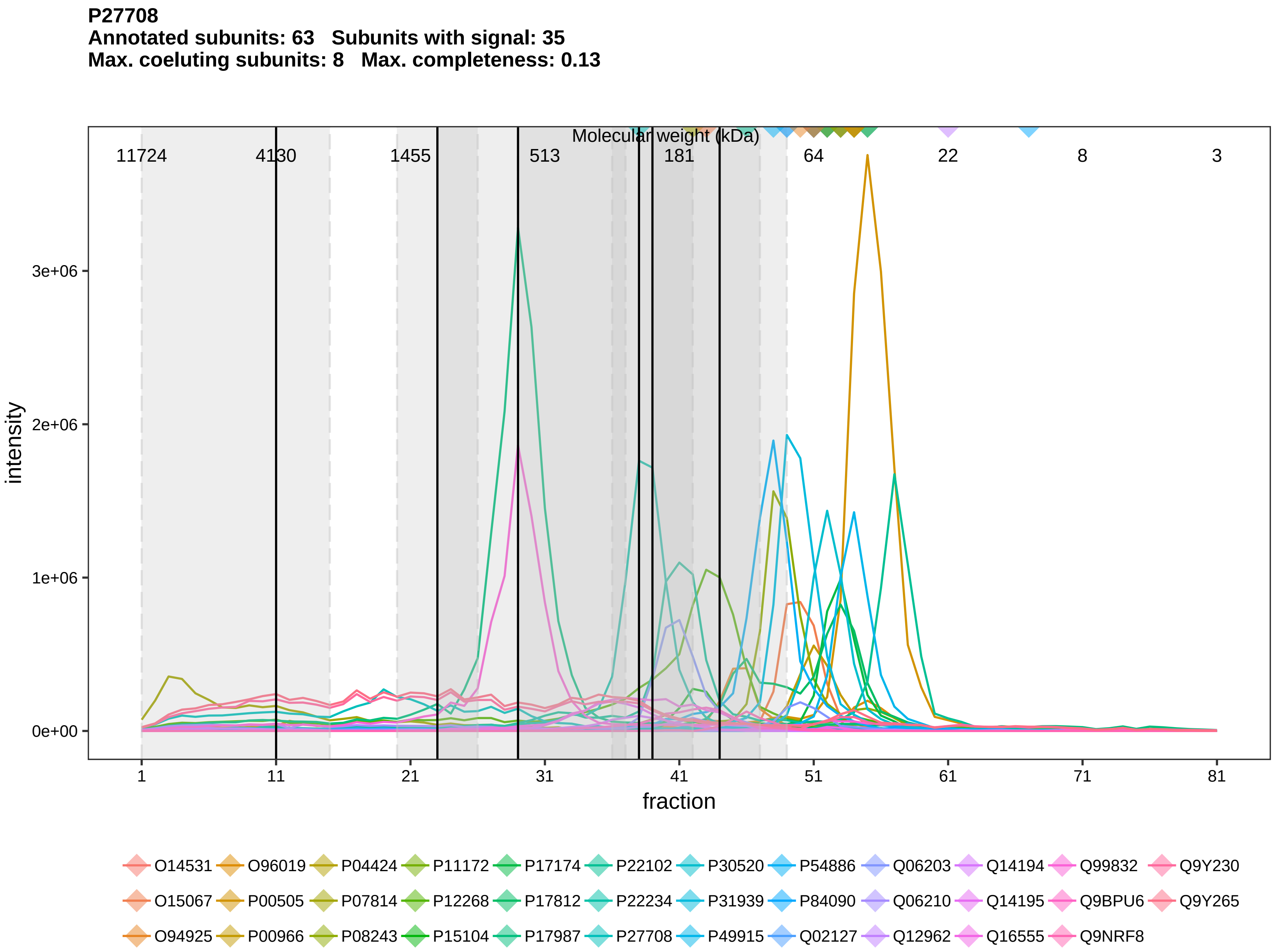
P27708
Annotated subunits: 63 Subunits with signal: 35
Max. coeluting subunits: 8 Max. completeness: 0.13
Molecular weight (kDa)
11724
4130
1455
513
181
64
22
8
3
intensity
3e+06
2e+06
1e+06
0e+00
fraction
1
11
21
31
41
51
61
71
81
O14531
O96019
P04424
P11172
P17174
P22102
P30520
P54886
Q06203
Q14194
Q99832
Q9Y230
O15067
P00505
P07814
P12268
P17812
P22234
P31939
P84090
Q06210
Q14195
Q9BPU6
Q9Y265
O94925
P00966
P08243
P15104
P17987
P27708
P49915
Q02127
Q12962
Q16555
Q9NRF8
